# *Tamarix articulata* Inhibits Cell Proliferation, Promotes Cell Death Mechanisms and Triggers G_0_/G_1_ Cell Cycle Arrest in Hepatocellular Carcinoma Cells

**DOI:** 10.17113/ftb.59.02.21.6904

**Published:** 2021-06

**Authors:** Abdullah M. Alnuqaydan, Bilal Rah

**Affiliations:** Department of Medical Biotechnology, College of Applied Medical Sciences, Qassim University, P.O. Box 6666, 51452 Buraidah, Saudi Arabia

**Keywords:** *Tamarix articulata*, autophagy, apoptosis, cell cycle, antiproliferative activity

## Abstract

**Research background:**

From ancient times plants have been used for medicinal purposes against various ailments. In the modern era, plants are a major source of drugs and are an appealing drug candidate for the anticancer therapeutics against various molecular targets. Here we tested methanolic extract of dry leaves of *Tamarix articulata* for anticancer activity against a panel of hepatocellular carcinoma cells.

**Experimental approach:**

Cell viability of hepatocellular carcinoma cells was determined by 3-(4,5-dimethylthiazol-2-yl)-2,5-diphenyltetrazolium bromide (MTT) assay after a dose-dependent treatment with the extract of *T. articulata.* Phase-contrast microscopy and 4՛,6-diamidino-2-phenylindole (DAPI) staining served to analyse cellular and nuclear morphology. Immunoblotting was performed to determine the expression of proteins associated with autophagy, apoptosis and cell cycle. However, flow cytometry was used for the quantification of apoptotic cells and the analysis of cells in different phases of the cycle after the treatment with various doses of *T. articulata*. Additionally, acridine orange staining and 2՛,7՛-dichlorofluorescein diacetate (DCFH-DA) dye were used to analyse the quantification of autophagosomes and reactive oxygen species.

**Results and conclusion:**

Our results demonstrate that *T. articulata* methanolic extract exhibits promising antiproliferative activity with IC_50_ values (271.1±4.4), (298.3±7.1) and (336.7±6.1) µg/mL against hepatocellular carcinoma HepG2, Huh7D12 and Hep3B cell lines, respectively. Mechanistically, we found that *T. articulata* methanolic extract induces cell death by activating apoptosis and autophagy pathways. First, *T. articulata* methanolic extract promoted autophagy, which was confirmed by acridine orange staining. The immunoblotting analysis further confirmed that the extract at higher doses consistently induced the conversion of LC3I to LC3II form with a gradual decrease in the expression of autophagy substrate protein p62. Second, *T. articulata* methanolic extract promoted reactive oxygen species production in hepatocellular carcinoma cells and activated reactive oxygen species-mediated apoptosis. Flow cytometry and immunoblotting analysis showed that the plant methanolic extract induced dose-dependent apoptosis and activated proapoptotic proteins caspase-3 and PARP1. Additionally, the extract triggered the arrest of the G_0_/G_1_ phase of the cell cycle and upregulated the protein expression of p27/Kip and p21/Cip, with a decrease in cyclin D1 expression in hepatocellular carcinoma cells.

**Novelty and scientific contribution:**

The current study demonstrates that *T. articulata* methanolic extract exhibits promising anticancer potential to kill tumour cells by programmed cell death type I and II mechanisms and could be explored for potential drug candidate molecules to curtail cancer in the future.

## INTRODUCTION

Hepatocellular carcinoma (HCC) is the predominant primary cancer in most countries and seventh most frequent cancer across the globe (*1*). HCC is the second most lethal cancer-associated mortality in the world, with an annual rate of around 1.2 million deaths, mainly in the under-developing and developing countries of East Asia and Africa (*2*). The incidence of HCC varies according to gender, age, ethnicity and geographical distribution. The majority (more than 80%) of HCC cases occur in African and East Asian countries, with an incidence rate of more than 20 people in 100 000 individuals. HCC rarely appears below the age of 40 and males are at higher risk than females (*3*). Several etiological factors that have a casual association with HCC are chronic viral infections such as hepatitis B virus (HBV) or hepatitis C virus (HCV), frequent exposure to aflatoxin B1, liver cirrhosis due to alcohol abuse, and non-alcoholic fatty liver (*4*). Patients with early disease are often asymptomatic and consequently diagnosed at a late stage when the disease is untreatable (*5*).

At the molecular level, HCC is complex and heterogeneous due to several key mutations. These mutations cause aberrant modulations of key proteins that result in the deregulation of crucial signalling pathways (*6*, *7*). Owing to many genetic mutations in the HCC and the deregulation of many signalling pathways, HCC is often resistant to the current regiment of therapeutic modalities (*8*). The therapeutic options are surgical resection and are often more successful if the malignancy is diagnosed at an early stage of the disease (*9*). Although liver transplantation is the best effective therapeutic approach to deal with HCC; only around 5% of patients can benefit from this approach because of histocompatibility and organ availability issues (*10*). Except transplantation, other therapeutic options are chemotherapy and radiotherapy. Regrettably, HCC resists most cytotoxic drugs. Among chemotherapeutic drugs, sorafenib, a kinase inhibitor, is the drug of choice against HCC and increases patient’s survival time for only a few months (*11*).

For the last two decades, various novel synthetic chemotherapeutic drugs have been used in clinical settings to treat cancer, but still not much success has been achieved due to considerable cost of development and off-target deleterious effects on normal cells, which causes serious adverse effects like gastrointestinal upset, suppression of immune system, and the development of drug resistance, thereby leading to failure of these drugs (*12*). Thus, identification and evaluation of plant-based novel extracts or compounds are seemingly important.

Natural products obtained from the plant kingdom have received great attention for promising chemotherapeutic potential and are the prime source of medicines for other ailments. Owing to their structural complexity, natural products can hit multiple targets at various stages of tumourigenesis and associated inflammation (*13*). This provides evidence that natural products are promising anticancer molecules to target deregulated signalling pathways at multiple sites to curtail cancer. More than 60% of anticancer drugs come from plant-based natural products or their derivatives (*14*). At present, around 3000 plants across the globe have been reported to have anticancer potential. Globally, around 10 to 40% of plant-derived products are used for the treatment of cancer and are expected to reach 50% in Asia (*15*). Therefore, there is a continuous demand for search and development of novel and effective drugs from natural sources (plant sources) which specifically kill tumour cells with fewer undesirable effects against normal cells.

Plant-based extracts or natural compounds derived from plant extracts with chemopreventive activity are a vital source of drugs against many diseases including cancer (*16*). Approximately more than 50% of the current regiment of the Food and Drug Administration (FDA)-approved drugs are derived from plants (*17*). Yet, there are numerous plants of various species that could have the potential to exhibit pharmacological activities and can be an important source of novel compounds for future therapeutics with great efficacy against tumour cells and fewer toxicity issues against normal human cells. Intending to search for novel compounds with less deleterious effects, we focused on natural medicines. One of the plants from the deserts of Saudi Arabia containing a variety of secondary metabolites that could be used for the production of anticancer therapeutics is *Tamarix articulata* (*18*). The plant belongs to the family *Tamaricaceae* and is a halophytic plant growing in extremely harsh and arid conditions. Traditionally, the plant is used to treat various ailments such as gastrointestinal, skin and heart diseases and other ailments (*19*). However, recent evidence suggests that owing to the presence of various polyphenolic compounds, *T. articulata* exhibits some promising pharmacological activities such as antioxidant, antidiabetic, hypolipidemic, antibacterial and hepatoprotective activities (*20*-*24*). The major chemical constituents of the *T. articulata* dry leaf extract are quinic acid, gallic acid, kaempferol, quercetin, tamarixetin, epicatechin gallate and epiafzelechin. These phytochemicals are responsible for pharmacological properties including antioxidant, antibacterial, anticancer, antidiabetics and hepatoprotective activities (*18*). Crude extracts of the plants in other parts of the world have been reported to exhibit anticancer activities by inhibiting cell viability in various types of cancer cells (*17*). Our recent study (*20*) reveals that methanolic extracts of all *T. articulata* parts (root, stem, dry and fresh leaves) collected from the Qassim region of Saudi Arabia have promising antioxidant activity due to the presence of polyphenolic and flavonoid compounds. Additionally, we documented that all methanolic extracts of different plant parts exhibit potential anticancer effect; however, the data were preliminary and without any mechanistic study. Therefore, in the current study, we evaluated the antiproliferative activity of the methanolic extract of dry leaves of *T. articulata* against a panel of HCC cells (HepG2, Hep3B and Huh7D12) in an attempt to better understand the underlying anticancer mechanism. We observed that the plant methanolic extract simultaneously induces apoptosis and autophagy in HCC (Hep2G) cells. Additionally, the plant methanolic extract triggered the arrest of the G_0_/G_1_ phase of the cell cycle and increased the protein expression of cyclin-dependent kinase inhibitor 1B (p27/Kip), encoded by the *CDKN1B* gene and cyclin-dependent kinase inhibitor 1 (p21/Cip), encoded by the *CDKN1A* gene, with a decrease in the expression of cyclin D1 in Hep2G cells. Together, these results proved that *T. articulata* medicinal plant can be explored as a potential drug candidate to curtail cancer in near future.

## MATERIALS AND METHODS

### Collection of plant material and extract preparation

The plant material (leaves of *Tamarix articulata*) was collected in August 2019 from desert regions of Qassim province of the Kingdom of Saudi Arabia. The leaves were air dried in the shade to remove moisture. The methanolic extract of dry leaves was prepared as per the standard protocol mentioned in our previous publication (*21*).

### Cell culture and treatments

Hepatocellular carcinoma (HCC) cells (HepG2, Hep3B and Huh7D12) and normal transformed cells THLE-2 were procured from the American Type Culture Collection (ATCC; Manassas, VA, USA). All the procured cell lines were cultured in the required medium (minimum essential medium; Thermo Fisher Scientific, Waltham, MA, USA) and maintained under sterile conditions at 37 °C in a 5% CO_2_ incubator (New Brunswick Scientific, Maldon, UK). Penicillin-streptomycin (pen-strep, 1%; Thermo Fisher Scientific) and 10% foetal bovine serum from Gibco (Dublin, Ireland) were added to the growth medium to supply essential growth factors for the proper growth of the cells. The cells were periodically evaluated for mycoplasma contamination.

### Preparation of plant stock solution

The stock solution of 10 mg/mL dry leaf methanolic extract was made in dimethyl sulfoxide aliquoted and stored at -20 °C in sterile microcentrifuge (1.5 mL) tubes. At the time of experiment, stock concentration was diluted serially in culture medium to obtain the working concentration in a range of 10-10 000 µg/mL for the treatment of HCC (HepG2, Hep3B and Huh7D12) cells.

### Chemicals and antibodies

Propidium iodide, rapamycin, camptothecin, 3-(4,5-dimethylthiazol-2-yl)-2,5-diphenyltetrazolium bromide (MTT), hydrogen peroxide, 4’,6-diamidino-2-phenylindole (DAPI), staurosporine, 2′,7’-dichlorofluorescein diacetate (DCFH-DA), dimethyl sulphoxide (DMSO), FITC-conjugated annexin V, acridine orange, Tris-buffered saline with 0.1% Tween^®^ 20 detergent and *N*-acetylcysteine came from Sigma-Aldrich, Merck (St. Louis, MO, USA). The required primary antibodies (cyclin D1, β-actin, p27, p21, MAPLC3I/II, SQSTM1/P62, PARP1 and caspase-3) were from Cell Signaling Technology (Leiden, The Netherlands). The enzyme-labelled mouse and rabbit IgG secondary antibodies were procured from Santa Cruz Biotechnology (Dallas, TX, USA).

### Cell proliferation/viability assay

The cell viability was determined by the most commonly used MTT assay as per the standard protocol (*25*). After trypsinization of HCC cells (HepG2, Hep3B, Huh7 and THLE-2) from the primary cell culture flask, the cells were uniformly suspended in culture medium and distributed in 96-well plate at a density of 2·10^3^ cells per well. After overnight incubation, the cells were exposed to various doses of plant methanolic extract and DMSO control for 24 h. The plated cells were flooded with 20 µL of 2.5 mg/mL MTT dye solution at 37 °C for 3-4 h. The MTT dye interacts with succinyl dehydrogenase of live mitochondria to form formazan crystals. The purple colour formation developed after dissolving the formazan crystals in DMSO. Subsequently, the absorbance of the coloured solution was measured at 570 nm with the help of the multi-plate reader. The number of viable cells was analysed by GraphPad Prism v. 5.0 software (*26*) to calculate the percentage of cell proliferation of the treated group compared to the DMSO control group.

### Apoptosis detection using flow cytometry

Annexin V-FITC apoptosis detection kit was employed to analyse apoptosis using flow cytometry (*27*). In brief, 5·10^5^ HCC (HepG2) cells were harvested and plated in each well of the 6-well plate and allowed to adhere to the surface properly overnight. The cells were exposed to various doses of plant methanolic extract in the presence of untreated control for 24 h under sterile conditions at 37 °C and 5% CO_2_. Next, the cells were harvested, washed with ice-cold phosphate-buffered saline (PBS) 3 times, and resuspended in binding buffer solution followed by the addition of 5 µL each of FITC-conjugated annexin V and propidium iodide for 10-15 min at 4 °C in the dark. After that, incubation samples were analysed using flow cytometry (BD FACS Calibur^TM^; BD Bioscience, Franklin Lakes, NJ, USA) to quantify the apoptotic cell population.

### Cell cycle analysis

Briefly, 5·10^5^ HepG2 cells were harvested and plated in each well of the 6-well plate and allowed to adhere properly (*28*). The cells were exposed to various concentrations (125, 250 and 500 mg/mL) of the plant methanolic extract and untreated control at 37 °C in a 5% CO_2_ incubator for 24 h. The treated cells were harvested, washed and fixed in 70% ice-cold ethanol overnight at 4 °C. The next day, the cells were processed for cell cycle analysis by incubation with RNase (100 μg/mL) for 30 min at 37 °C and stained with propidium iodide (50 μg/mL) in the dark for another 30 min at 4 °C. The cell cycle analysis was done by using the BD^TM^ LSRII flow cytometry system (BD Bioscience) to analyse cells in different phases of their cycle.

### Phase-contrast microscopy

HCC (HepG2) cells (5·10^5^) were seeded on coverslips in 30-mm Petri dishes and were exposed to different concentrations of plant methanolic extract for 24 h (*29*). After completion of the treatment, the cells were processed for phase-contrast microscope LSM-510 (Carl Zeiss, Munich, Germany) to detect the phenotypic changes in the morphology of the treated cells.

### Immunoblotting

Briefly, 0.5·10^6^ HepG2 cells were harvested and seeded in each well of the 6-well plate overnight to adhere properly as per the standard protocol (*16*). The plated HCC (HepG2) cells were treated with different doses of plant methanolic extract (125, 250 and 500 mg/mL) and control DMSO for 24 h. After the treatment, the cells were harvested, washed with PBS and resuspended in cell lysis buffer. The resuspended cells were constantly vortexed for 10 s and the solution was kept in ice for at least 2 min, and the step was repeated at least five times. The obtained cell lysis solution was subjected to centrifugation (12 000×*g*; 5810 R; Eppendorf, Chennai, India) at 4 °C for 10 min. The obtained supernatant was collected in separate autoclaved microtubes, followed by protein estimation using standard Bradford method (*30*). An equal amount of protein sample (30 µg) obtained from various treatments was loaded in each well of pre-cast SDS-PAGE gel and the gel was allowed to resolve based on molecular mass at a standard voltage and current. Properly resolved proteins of the SDS-PAGE gel were transferred onto the polyvinylidene fluoride (PVDF) membrane (Millipore, Darmstadt, Germany) incubated with a 5% blocking solution containing fat-free milk in Tris-buffered saline with 0.1% Tween^®^ 20 detergent (TBST) to block non-specific antigen-binding sites. The PVDF membrane was incubated with the primary antibody at the desired dilution at room temperature for 3-4 h or overnight at 4 °C to ensure proper binding with respective protein/antigen. After the completion of incubation, the PVDF membrane was gently washed with TBST at least five times and was again probed with enzyme-tagged IgG secondary antibody for 1 h at 37 °C. The membrane was again gently washed with TBST at least five times. The membrane was flooded and incubated in enhanced chemiluminescence plus substance to spot the presence of the protein with the help of x-ray film or with ChemiDoc imaging system (Bio-Rad, Hercules, CA, USA).

### Reactive oxygen species activity determination

Reactive oxygen species (ROS) activity was determined by commonly used DCFH-DA dye which is oxidised to green, fluorescent dichlorofluorescein (DCF) as per the standard protocol (*31*). Briefly, 10^5^ HCC (HepG2) cells were harvested after trypsinization and plated in each well of a 6-well plate overnight to attach to the surface properly. The plated cells were exposed to various doses of plant methanolic extract in the presence and absence of *N*-acetylcysteine (5 mM) (Sigma-Aldrich, Merck), untreated control and positive control (H_2_O_2_) at 37 °C for 24 h. The treated cells in each well were gently washed with ice-cold PBS and incubated with DCFH-DA dye for 30 min at 37 °C in a 5% CO_2_ incubator (New Brunswick^TM^ S41i; Eppendorf) for staining. Subsequently, stained cells were photographed under a fluorescent microscope (Eclipse E600; Nikon, Tokyo, Japan) and the plates were analysed by a fluorescence microplate reader (Infinite F50; Tecan, Männedorf, Switzerland) at 488 nm excitation and 525 nm emission wavelengths.

### Autophagosome staining

The detection of autophagosomes by microscopy was done according to standard protocol (*31*). Briefly, 10^5^ HCC (HepG2) cells were harvested after trypsinization and plated in each well of a 6-well plate overnight to attach to the surface properly. The plated cells were exposed to various doses (125, 250 and 500 µg/mL) of plant methanolic extract, untreated control, and positive control (rapamycin, *c*=100 nM) at 37 °C for 24 h. Next, the cells containing plant methanolic extract in culture medium were removed and fresh medium containing acridine orange (1 mg/mL) was added and incubated for 15 min at 37 °C in 5% CO_2_. The plated acridine orange-stained cells were analysed under a fluorescent microscope. They were bright red due to low or acidic pH in the autophagosomes. Cells from many fields were counted until 100 cells were reached to determine the percentage of cells with promoted autophagosome formation.

### Nuclear morphology determination by DAPI staining

Nuclear morphology was analysed by 4’,6-diamidino-2-phenylindole (DAPI) staining (*32*). Briefly, 5·10^4^ HCC (HepG2) cells were seeded and plated in the 8-well chamber slide and allowed to adhere properly to the surface of the chamber slide. Next morning, HCC (HepG2) cells were treated with different doses of plant methanolic extract, untreated control, and positive control (camptothecin, *c*=2 µM) for 24 h. Cells were rinsed with cooled PBS, followed by fixing and permeabilisation with 4% paraformaldehyde and Triton X-100, respectively. Finally, the fixed cells were mounted with Ultracruz mounting medium and were analysed by a LSM-510 microscope (Carl Zeiss) for the detection of nuclear morphology.

### Statistical analysis

All the experimentations were executed (*N*=3). The statistical analysis of all experiments was performed using GraphPad Prism software (*26*) from three independent experiments. All the obtained results represent the mean value ± standard error of the mean (SEM), calculated and processed by one-way ANOVA. The p-value equal to or less than 0.05 was considered significant.

## RESULTS AND DISCUSSION

**Fig. 1:**
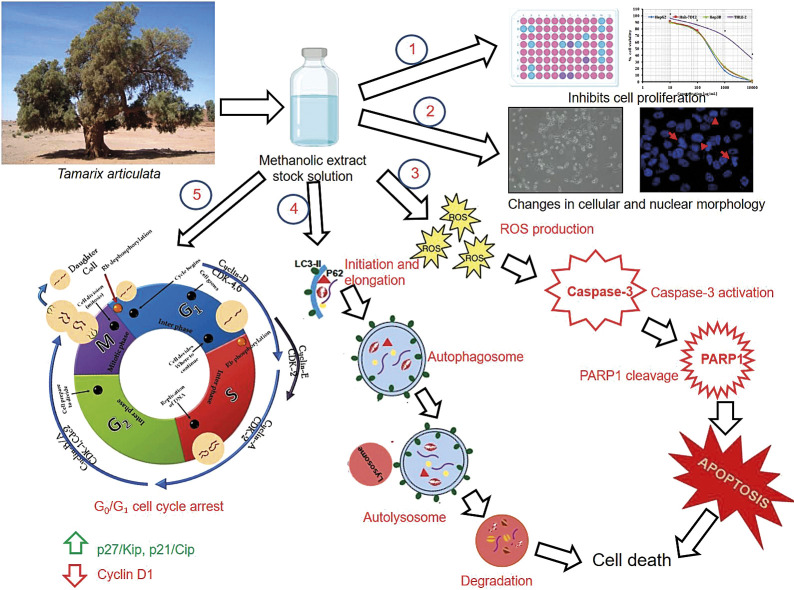
The schematic diagram of the methanolic extract of *Tamarix articulata* dry leaves exerts the antiproliferative effect by simultaneous induction of cell death by two biologically conserved processes: autophagy by the conversion of LC3I to LC3II followed by the degradation of SQSTM1/P62 and apoptosis that is associated with the activation of casapase-3 and PARP1 cleavage in HepG2 cells. Furthermore, the methanolic extract of dry leaves triggers cell cycle arrest at G_0_/G_1_ phase by upregulating the regulatory proteins p27/Kip and p21/Cip with concomitant reduction in cyclin D1 in HCC cells. ROS=reactive oxygen species

The current study demonstrated for the first time that the methanolic extract of *Tamarix articulata* dry leaves exhibits cytotoxic potential and promotes simultaneous induction of autophagy and apoptosis in HCC (HepG2) cells. A mechanistic study reveals that upon treatment with various doses of the plant methanolic extract, HCC (HepG2) cells augment autophagic cell death by promoting the formation of autophagosomes, which were analysed by acridine orange staining. Furthermore, the immunoblotting demonstrates the steady conversion of microtubule-associated protein 1A/1B-light chain 3-I (LC3I) to microtubule-associated protein 1A/1B-light chain 3-II (LC3II) with gradual degradation of sequestosome-1 (SQSTM1/p62) upon treatment with various doses of the plant extract. Moreover, we observed a significant induction of apoptosis, which was supported by activation of apoptosis marker proteins caspase-3 and PARP1 at higher doses of the plant methanolic extract. DAPI staining, phase contrast microscopy and elevated levels of ROS further confirmed the cell killing by apoptosis. Additionally, the plant methanolic extract triggered cell cycle arrest at the G_0_/G_1_ phase and increased the expression of cell cycle checkpoint proteins p27/Kip and p21/Cip with a decrease in the expression of cyclin D1 in the cells treated with various doses of the plant methanolic extract (Fig. 1). Together these results suggest that the medicinal plant *T. articulata* can be explored as a potential drug candidate to curtail cancer in the future.

**Fig. 2:**
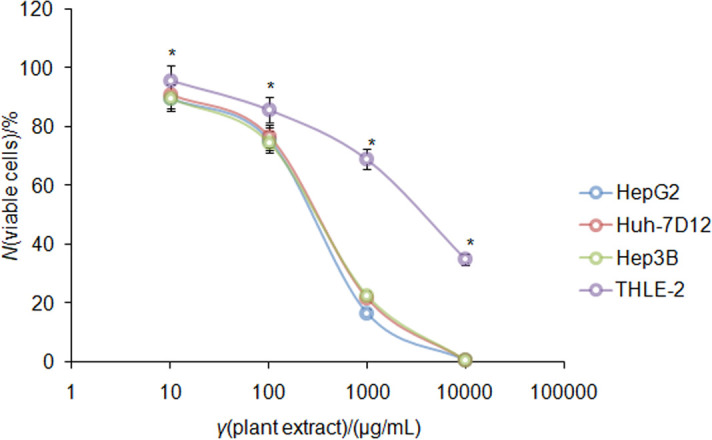
Antiproliferative effect of methanolic extract of *Tamarix articulata* dry leaves against hepatocellular carcinoma cells (HepG2, Huh-7D12 and Hep3B, and transformed cell line THLE-2), confirmed by the MTT assay. Data are presented as the mean value±standard error (S.E.), *N*≥3, *p<0.05

Previous reports demonstrated that numerous plant extracts exhibit an antiproliferative potential against a wide range of cellular models of cancer (*33*, *34*). *T. articulata* methanolic extract reveals several pharmacological properties like anti-inflammatory, hepatoprotective, antioxidative and antiproliferative activity by modulating miR-1275 in hepatocellular carcinoma cells (*18*, *20*). Thus, we sought to examine the *in vitro* inhibitory activity of the plant methanolic extract against the HCC cell (HepG2, Huh7, Hep3B and THLE-2) proliferation. We observe that the plant methanolic extract started attenuation of growth kinetics of HCC (HepG2, Huh7, Hep3B and THLE-2) cells at an incredibly low dose of 10 µg/mL. It was observed that the IC_50_ value was (271.0±4.4), (298.0±7.1) and (336.0±6.1) µg/mL against HepG2, Huh7D12 and Hep3B cell lines, respectively (Fig. 2 and Table 1). Interestingly, when the inhibitory effect on the growth of transformed normal THLE-2 cells was evaluated, we observed that 50% population of the cells died at (3641.0±23.7 µg/mL) of the plant methanolic extract (Fig. 2 and Table 1), which is a significantly high dose of the plant methanolic extract compared to IC_50_ dose against HCC (HepG2, Huh7, Hep3B and THLE-2) cells. This indicates that the plant methanolic extract not only curtails the cellular growth kinetics of HCC cells specifically, but also displays safe cytotoxicity potential against normal cells. Together, our results suggest that the methanolic extract of dry leaves of halophytic *T. articulata* plant displays potential cytotoxic effect against a panel of HCC cells and leaving normal transformed liver cells with weaker cytotoxic effect.

**Table 1:** IC_50_ value of the methanolic extract of *Tamarix articulata* dry leaves against a panel of hepatocellular carcinoma cells (HepG2, Huh-7D12 and Hep3B) and transformed normal hepatic cells (THLE-2)

Cell line	IC_50_/(µg/mL)
HepG2	271.1±4.4
Huh-7 D12	298.3±7.1
Hep3B	336.7±6.1
THLE-2	3641±23

Data are presented as the mean value±standard error (S.E.), *N*≥3, p<0.05

**Fig. 3:**
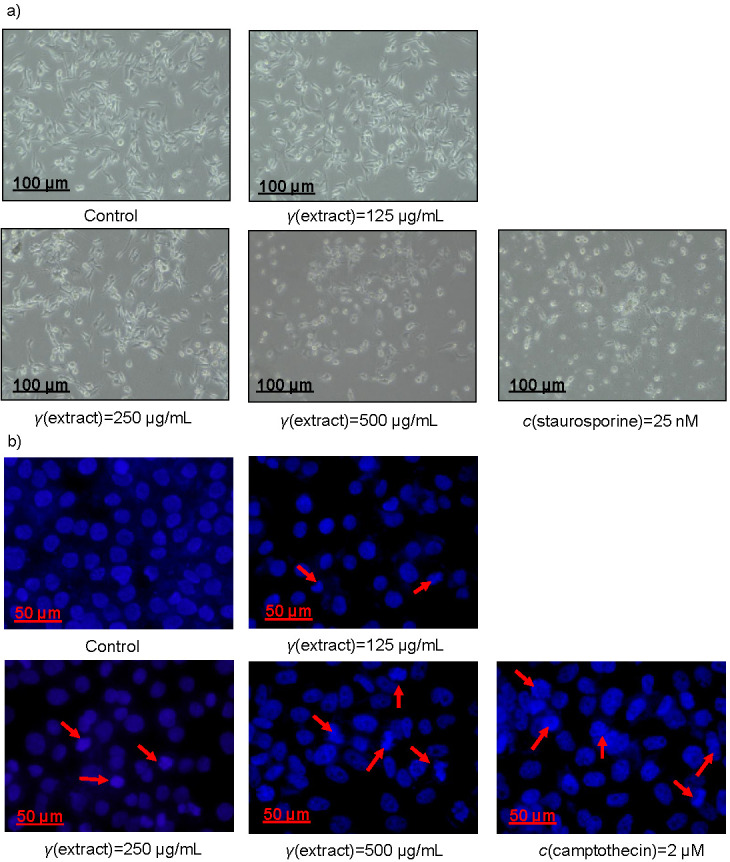
Induction of HepG2 cell death by methanolic extract of *Tamarix articulata* dry leaves: a) HepG2 cells treated with various concentrations of the extract, positive control (c(staurosporine)=25 nM) and untreated control (DMSO). The images were taken by a microscope inbuilt with a camera; scale bar: 100 µm, magnification: 20×, and b) images of 4’,6-diamidino-2-phenylindole (DAPI) stained cells after treatment with the extract, positive control (*c*(camptothecin)=2 µM) and untreated control (DMSO). Scale bar: 50 µm, magnification: 20×. DMSO=dimethyl sulphoxide

Next, the microscopic analysis indicated that a substantial number of dead, floating tumour cells were observed at higher doses of the plant methanolic extract than untreated control (Fig. 3a), indicating that the percentage of cell death in groups treated with the plant extract was significantly higher than with the DMSO control. Furthermore, cell killing by the plant methanolic extract was also confirmed by the nuclear morphology of DAPI stain, which revealed the change in the membrane blebbing and nuclear morphology, which are the hallmarks of apoptosis (Fig. 3b). The above results demonstrate that the plant methanolic extract induces a potent antiproliferative effect on the cellular models of HCC (HepG2, Huh7, Hep3B and THLE-2).

**Fig. 4:**
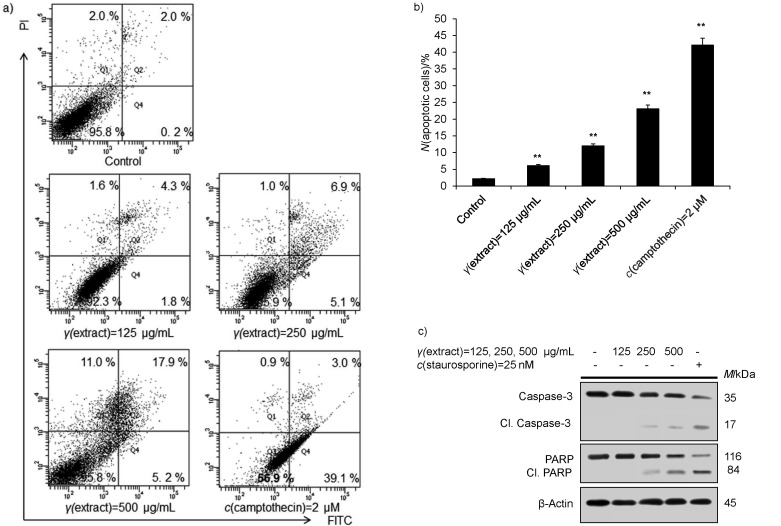
Induction of apoptosis in HepG2 cells by methanolic extract of *Tamarix articulata* dry leaves: a) quantification of the apoptotic population by flow cytometry of HepG2 cells after 24-hour treatment with different concentrations of the extract, positive control (*c*(staurosporine)=25 nM) and untreated control (DMSO); Q1 represents necrotic cell population, Q2 represents early apoptotic cell population, Q3 represents normal cells, whereas Q4 represents late apoptotic cell population, b) percentage of apoptotic HepG2 cells in the above-mentioned experiment. Data are expressed as the mean value±S.E., *N*≥3, **p<0.01, and c) protein expression of apoptosis-activated proteins (total caspase-3, cleaved caspase-3, total PARP1, cleaved PARP1, and β-actin as an internal control) of HepG2 cells after 24-hour treatment with different concentrations of the extract, positive control (*c*(staurosporine)=25 nM) and untreated control (DMSO). DMSO=dimethyl sulphoxide, PI=propidium iodide, FITC=fluorescein isothiocyanate

Badmus *et al.* (*35*) suggested that *Holarrhena floribunda* extract displayed an antiproliferative activity against tumour cells and triggered cell cycle arrest by inducing ROS-mediated apoptosis induction. Similarly, Kowalczyk *et al.* (*36*) showed that *Menyanthes trifoliata* L. extract significantly inhibited the growth of glioma cells by attenuating the expression of anti-apoptotic proteins, triggered arrest of the G_2_/M phase of cell cycle, activated mitochondrion-dependent apoptosis and augmented the expression of apoptosis proteins Bax and caspase-3. Various studies have revealed that natural compounds obtained from plants have a promising apoptosis-inducing ability in cancer cells (*37*–*39*). Mechanistically, apoptosis induced by natural compounds disrupts the association of antiapoptotic protein Bcl-2 from the proapoptotic proteins, thereby activating a cascade of proapoptotic proteins (*40*). Plant-based extracts and derived compounds have been documented to activate cell death mechanisms by activating cellular caspases, promote intrinsic apoptosis by releasing cytochrome C, induce ROS production and activation of 5' adenosine monophosphate-activated protein kinase (AMPK) which ultimately leads to activation of cell death mechanisms in tumour cells (*41*, *42*). Thus, we wanted to evaluate the apoptotic potential of *T. articulata* methanolic extract against HCC (HepG2) cells. Therefore, we exposed HepG2 cells to different doses of the plant methanolic extract for 24 h and untreated control. With flow cytometry, we observed that a substantial cellular population of HepG2 cells underwent apoptosis (23.1%) at a higher dose of the plant methanolic extract (500 μg/mL) than with positive control staurosporine (25 nM) (42.1%) (Fig. 4a and Fig. 4b). To further confirm the activation of apoptosis, we performed immunoblotting analysis of HepG2 cells treated with the plant methanolic extract in a concentration-dependent manner (125, 250 and 500 µg/mL). Our results suggest that higher concentrations of the plant methanolic extract activate apoptosis-promoting proteins by inducing prominent cleavage of caspase-3 and PARP1 when compared to untreated control (Fig. 4c).

**Fig. 5:**
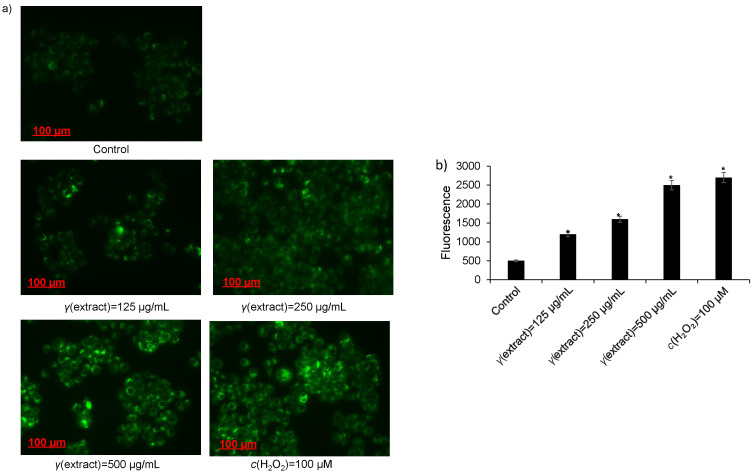
Induction of ROS production in HepG2 cells by methanolic extract of *Tamarix articulata* dry leaves: a) fluorescent images of 2′,7’-dichlorofluorescein diacetate (DCFH-DA) stained cells after 24-hour treatment with different concentrations of the extract, positive control (H_2_O_2_) and untreated control (DMSO). Scale bar: 100 µm, magnification 20×, and b) the fluorescence intensity of DCF cells treated with the extract, positive control (H_2_O_2_) and untreated control (DMSO). Data are expressed as the mean value±S.E., *N*≥3, *p<0.05. DMSO=dimethyl sulphoxide, DCF=dichlorofluorescein

ROS and associated peroxidases play a key role in the regulation of cell death in multiple cancer cell types. Accumulated evidence revealed that elevation of ROS by natural products is the major driver of the activation of cancer cell death pathways. In the recent past, reports have suggested that chemotherapeutic agents induce apoptotic cell death by elevating ROS production (*31*). To examine whether the plant methanolic extract could also induce ROS production in HepG2 cells, we exposed HepG2 cells to various doses of the plant extract in the presence of ROS activator H_2_O_2_ (positive control, 100 μM) or untreated control for 24 h and stained the cells with DCFH-DA dye after the treatment. DCFH-DA dye easily passes through the membrane by diffusion and is deacetylated with cellular esterases to non-fluorescent compound. The non-fluorescent compound is then oxidised to produce green colour compound 2',7'-dichlorofluorescein in the presence of ROS. Intriguingly, we observed that the plant extract promotes significant ROS production in a concentration-dependent manner as compared to untreated control. However, HepG2 cells exposed to positive control (H_2_O_2_, 100 μM) significantly promoted ROS production (Fig. 5). Together, these results demonstrate that the plant methanolic extract activates apoptosis-mediated proteins and promotes ROS production, which might have a critical role in the augmentation of apoptosis.

**Fig. 6:**
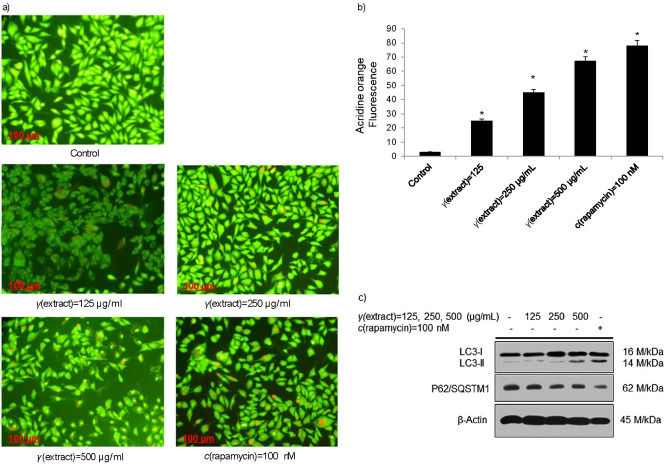
Induction of autophagy in HepG2 cells by methanolic extract of *Tamarix articulata* dry leaves: a) fluorescent images of the acidic vesicular formation or autophagosome formation stained by acridine orange (*γ*=1 mg/mL) after a 24-hour treatment with different concentrations of the extract, positive control (*c*(rapamycin)=100 nM) and untreated control (DMSO). Scale bar: 100 µm, magnification: 20×, b) percentage of acridine orange-stained HCC cells upon treatment with different concentrations of the extract, positive control (*c*(rapamycin)=100 nM) and untreated control (DMSO). Data are expressed as the mean value±S.E., *N*≥3, *p<0.05, and c) expression of the conversion of autophagy proteins LC3I to LC3II and expression of p62/SQSTMI and β-actin (internal control) in HCC cells after 24-hour treatment with different concentrations of the extract, positive control (*c*(rapamycin)=100 nM) and untreated control (DMSO). DMSO=dimethyl sulphoxide

Autophagy is one of the important catabolic physiological processes that help clear the old and damaged cellular contents, recycle metabolites with the help of lysosomes and utilize them during energy crisis. It plays a dual role in cancer therapy and tumourigenesis. Accumulating evidence suggests that most natural products that exhibit antiproliferative activity induce autophagic cell death mode in tumour cells (*31*). A previous report revealed that in hepatocellular carcinoma cells, berberine suppresses hepatocellular carcinoma cells by inducing simultaneously mitochondrion-mediated apoptosis and autophagic cell death (*43*). Numerous reports suggest that plant-based natural compounds and extracts induce autophagy and kill tumour cells by various cell death mechanisms (*44*, *45*). Therefore, we sought to reveal whether *T. articulata* methanolic extract treatment could augment the induction of autophagy in HepG2 cells. To evaluate the effect of *T. articulata* methanolic extract, the HepG2 cells were plated overnight and exposed to various doses of the plant extract (Fig. 6a). After the completion of 24-hour incubation, the cells were stained with acridine orange. It is observable in Fig. 6b and Fig. 6c that although majority of the cells exposed to lower doses of the plant methanolic extract could not promote autophagy significantly and displayed acidic vesicular organelles (AVOs) in the cytoplasm, cells exposed to higher doses of the extract significantly induced AVOs, which appear bright red in the cytoplasm of HepG2 cells. Furthermore, immunoblotting of HepG2 cells at higher doses of the plant methanolic extract revealed a prominent conversion of LC3I to LC3II which indicates the maturation of autophagosome and induction of autophagy (Fig. 6c). To further evaluate the effect of the plant methanolic extract on the flux of autophagy in HepG2 cells, we observed the reduced expression with the low band intensity of sequestrosome 1 (P62) protein, which is a specific substrate of autophagy, at higher doses of the plant methanolic extract than at lower doses. Collectively, these results suggest that HepG2 cells exposed to the plant methanolic extract stimulate autophagic flux and augment cell death in tumour cells.

**Fig. 7:**
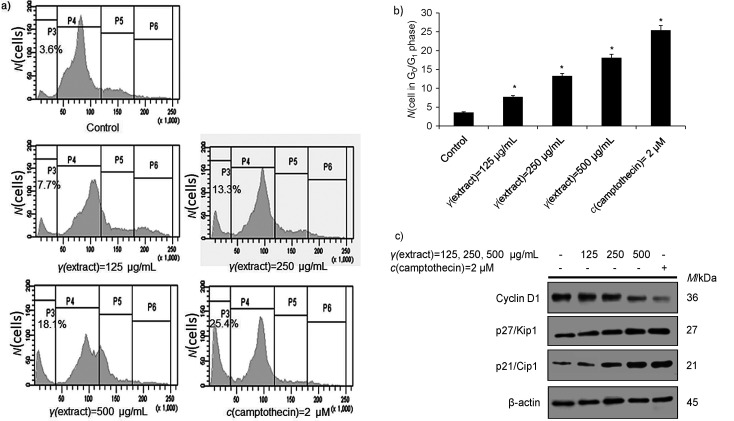
Induction of cell cycle arrest in G_0_/G_1_ phase by methanolic extract of *Tamarix articulata* dry leaves and modulation of regulatory proteins in HepG2 cells: a) quantification of cells in different stages of the cell cycle by flow cytometry after 24-hour treatment of HepG2 cells with different concentrations of the extract, positive control (*c*(camptothecin)=2 μM) and untreated control (DMSO), b) quantiﬁcation of G_0_/G_1_ phase cell cycle arrest in HepG2 cells after 24-hour treatment with different concentrations of the extract, positive control (*c*(camptothecin)=2 µM) and untreated control (DMSO). Data are expressed as the mean value±S.E., *N*≥3, *p<0.05, and c) expression profile of cell cycle regulation proteins (cyclin D1, p21/Cip1 and p27/Kip1) in immunoblotting analysis of HepG2 cells exposed to different concentrations of the extract, using β-actin as an internal control, positive control (*c*(camptothecin)=2 µM) and untreated control (DMSO). DMSO=dimethyl sulphoxide, P3=subG or G_0_, P4=G_1_/S, P5=G_2_, and P6=M

Accumulating evidence suggests that numerous phytochemicals derived from natural sources have been reported to exhibit antiproliferative activity by triggering cell cycle arrest, thereby promoting cell death *via* apoptosis (*45*, *46*). Tumour cell proliferation is primarily controlled by the cell cycle (*46*), which consists of four distinct progressive phases (subG1/G0, G1, S and G2/M) (*47*). Under normal circumstances, the progression of the cell cycle is tightly regulated by an activation cascade of cyclins, cyclin-dependent kinases (CDKs), and CDK inhibitors. However, in malignant cells, aberrant expression of cell cycle regulators attenuates the differentiation of cells that results in irregular cell growth. In the recent past, numerous reports have suggested that natural compounds have antiproliferative activity by triggering cell cycle arrest (*48*). Luo *et al*. (*49*) demonstrated that 6-gingerol (6-G) enhances the radiosensitivity of gastric cancer cells by the induction of apoptosis and cell cycle arrest. Additionally, Lin *et al.* (*50*) revealed that 6-G suppresses cell motility of colon cancer cells by inducing ROS, and upregulates p21/Cip and p27/Kip, thereby arresting cell cycle in colon cancer cells (*50*). To examine whether *T. articulata* methanolic extract could arrest the cell cycle, HepG2 cells were synchronised in a low serum-containing medium, and then advanced through various cell cycle phases in a serum-containing medium for at least 3 h. The cells were then treated with various doses (125, 250 and 500 µg/mL) of the plant methanolic extract for 24 h. Our results demonstrated that *T. articulata* methanolic extract arrested the cell cycle at G_0_/G_1_ phase in HepG2 cells. The quantification of cells at various cell cycle phases showed that a significant growth of cells (18.1%) was halted at the G_0_/G_1_ phase of the cell cycle when HepG2 cells were exposed to 500 µg/mL of plant extract as compared to untreated control (3.6%) and positive control 2 µM camptothecin (25.4%) (Fig. 7a and Fig. 7b). Next, we sought to examine the effect of the plant methanolic extract in the cell cycle regulatory proteins. Our immunoblotting results demonstrate a drastic reduction in cyclin D1 expression and a subsequent increase in the expression of p21/cip1 and p27/Kip1 proteins when HepG2 cells were exposed to higher doses of the plant methanolic extract (Fig. 7c). Collectively these results demonstrate that the plant methanolic extract induced G_0_/G_1_ phase cell cycle arrest and increased the expression of cell cycle checkpoint proteins p27/Kip1 and p21/Cip1 with a concomitant decrease in the expression of cyclin D1 in HepG2 cells.

## CONCLUSIONS

Our results document for the first time that the methanolic extract of dry leaves of *Tamarix articulata* exhibits cytotoxic activity and simultaneous activation of two cell death-associated processes in HepG2 cells: apoptosis and autophagy. Mechanistically, *T. articulata* induced autophagy, which was confirmed by prominent orange red colour of acidic autophagosomes and steady conversion of LC3I to LC3II with gradual degradation of SQSTM1/p62. Furthermore, the plant methanolic extract induced ROS-mediated apoptosis, which was confirmed by the activation of apoptosis marker proteins caspase-3 and PARP1. Additionally, the plant methanolic extract triggered the arrest of the G_0_/G_1_ phase in the cell cycle and increased the expression of cell cycle checkpoint proteins p27/Kip and p21/Cip with a decrease in the expression of cyclin D1 in the cells treated with various doses of plant methanolic extract. Collectively, these results prove that the medicinal plant *T. articulata* can be evaluated further as a prospective drug candidate compound to inhibit cancer.
